# Molecular interactions between *Neisseria meningitidis* and its human host

**DOI:** 10.1111/cmi.13063

**Published:** 2019-06-13

**Authors:** Mathieu Coureuil, Anne Jamet, Emmanuelle Bille, Hervé Lécuyer, Sandrine Bourdoulous, Xavier Nassif

**Affiliations:** ^1^ Inserm Institut Necker Enfants Malades U1151 Paris France; ^2^ Université de Paris UMR_S 1151 Paris France; ^3^ CNRS UMR 8253 Paris France; ^4^ Inserm, U1016 Institut Cochin Paris France; ^5^ CNRS UMR8104 Paris France; ^6^ Assistance Publique – Hôpitaux de Paris Hôpital Necker Enfants Malades Paris France

## Abstract

*Neisseria meningitidis* is a Gram‐negative bacterium that asymptomatically colonises the nasopharynx of humans. For an unknown reason, *N*. *meningitidis* can cross the nasopharyngeal barrier and invade the bloodstream where it becomes one of the most harmful extracellular bacterial pathogen. This infectious cycle involves the colonisation of two different environments. (a) In the nasopharynx, *N*. *meningitidis* grow on the top of mucus‐producing epithelial cells surrounded by a complex microbiota. To survive and grow in this challenging environment, the meningococcus expresses specific virulence factors such as polymorphic toxins and MDAΦ. (b) Meningococci have the ability to survive in the extra cellular fluids including blood and cerebrospinal fluid. The interaction of *N*. *meningitidis* with human endothelial cells leads to the formation of typical microcolonies that extend overtime and promote vascular injury, disseminated intravascular coagulation, and acute inflammation. In this review, we will focus on the interplay between *N*. *meningitidis* and these two different niches at the cellular and molecular level and discuss the use of inhibitors of piliation as a potent therapeutic approach.

## 1 INTRODUCTION

*Neisseria meningitidis* (the meningococcus) is a Gram‐negative bacterium that asymptomatically colonises the nasopharynx of 4% to 20% of humans (Christensen, May, Bowen, Hickman, & Trotter, 2010). For unknown reasons, *N*. *meningitidis* may invade the bloodstream where it becomes one of the most harmful extracellular bacterial pathogen. In some cases, meningococcemia will rapidly progress toward a septic shock leading in the worst cases to a *purpura fulminans*, an acute systemic inflammatory response associated with an intravascular coagulation and tissue necrosis (Bonazzi et al., 2018; Brandtzaeg & van Deuren, 2012; Capel et al., 2017; Lecuyer et al., 2018; Lecuyer, Borgel, Nassif, & Coureuil, 2017). Alternatively, *N*. *meningitidis* can be responsible for cerebrospinal meningitis after crossing the blood brain barrier (Coureuil, Lecuyer, Bourdoulous, & Nassif, 2017; Simonis & Schubert‐Unkmeir, 2016). The fatality ratio of meningococcal disease is 10–15% and may be up to 40% in case of *purpura fulminans*, with 20% of the survivors having permanent sequelae (Centers for Disease Control and Prevention (CDC), 2017).

The infectious cycle of *N*. *meningitidis* involves the colonisation of two different environments. (a) The natural habitat of *N*. *meningitidis* is the human nasopharynx from where bacteria are transmitted from person to person by aerosol droplets or direct contact with contaminated fluids. In the nasopharynx, *N*. *meningitidis* grows on the top of mucus‐producing epithelial cells surrounded by a complex microbiota. (b) During pathogenesis, meningococci have the ability to survive in the extra cellular fluids including blood and cerebrospinal fluid. The interaction of *N*. *meningitidis* with human endothelial cells is very unusual because it leads to the formation of typical microcolonies that extend overtime to ultimately fill in the microvessels. In this review, we will focus on the interplay between *N*. *meningitidis* and these two different niches at the cellular and molecular level.

## 2 COLONISATION OF THE NASOPHARYNX

The nasopharynx is lined by two types of epithelia: a stratified squamous epithelium that covers 60% of the nasopharynx and a columnar respiratory epithelium (Ali, 1965; Freeman & Kahwaji, 2018). Airway epithelial cells are covered by a 10–12‐μm thick airway surface liquid, itself composed of a low‐viscosity periciliary liquid layer and a high‐viscosity mucus facing the lumen and containing mucins polymers and antimicrobial peptides (Brandtzaeg, 2009; Cole, Dewan, & Ganz, 1999; Ganz, 2002). The airway mucus plays the role of a physical barrier (Fahy & Dickey, 2010; Lillehoj, Kato, Lu, & Kim, 2013). It also facilitates the elimination of particles or bacteria by the mucociliary clearance. Indeed, airway epithelial cells expressed cilia that beat synchronously to allow the clearance of the airway surface liquid (at a speed of 6.9 ± 0.7 mm/min [Hoegger et al., 2014]) from the lung to the pharynx and from the nose to the pharynx from where the mucus is swallowed (Paul et al., 2013). This mechanism is considered as the main defence against microorganisms and particles. Continuously drained from other compartments of the airways and unable to escape the mucus clearance, *N*. *meningitidis* is restrained to the nasopharyngeal mucosa at the crossroads of the two mucociliary escalators.

The airway mucus also possesses bacteriostatic and bacteriolytic properties that limit the growth of bacteria. (a) The mucus is a poor nutritive medium with low concentration of glucose (Garnett et al., 2016) and iron (Smith, Lamont, Anderson, & Reid, 2013), and (b) it is enriched in antimicrobial peptides/proteins (such as β‐defensins and cathelicidin LL‐37; Bals & Hiemstra, 2004), components of the complement system, and specific secretory immunoglobulin A that function by preventing attachment of bacteria to the components of the mucus itself (De Rose, Molloy, Gohy, Pilette, & Greene, 2018). The natural niche of meningococci is the human nasopharynx, and these bacteria are perfectly adapted to this niche. *N*. *meningitidis* is able to use several carbon sources including glucose, pyruvate, and lactate, the latter being found in the mucus in the millimolar range during inflammation of the airways (Bensel et al., 2011). It is also particularly well equipped to capture iron (see below #3). *N*. *meningitidis* expresses IgA protease and is extremely well equipped to survive against the innate immune system through expression of a polysaccharide capsule, the lipooligosaccharide (LOS) (Lo, Tang, & Exley, 2009), and the factor H binding protein (Seib et al., 2009; for an extended review, see Laver, Hughes, & Read, 2015). Meningococci also express the MtrCDE efflux pump that efflux antimicrobial peptides (Handing, Ragland, Bharathan, & Criss, 2018; Rouquette, Harmon, & Shafer, 1999). On the other hand, *N*. *meningitidis* is lack classical toxins, proteases, or type VI secretion system.

The mucus is highly colonised with a complex microbiota (Biesbroek et al., 2014; Cremers et al., 2014; Esposito & Principi, 2018; Santee et al., 2016; Wang et al., 2018) that does not seem to be favourable for colonisation by other bacterial species. Nonetheless, recent studies have revealed that families of polymorphic toxins and the presence of a prophage designated MDAΦ could play an essential role in the colonisation of the nasopharynx by meningococci, the former by allowing *N*. *meningitidis* to fight the local microbiota and the latter by increasing the colonisation rate.

### 2.1 Polymorphic toxins

**Figure 1:**
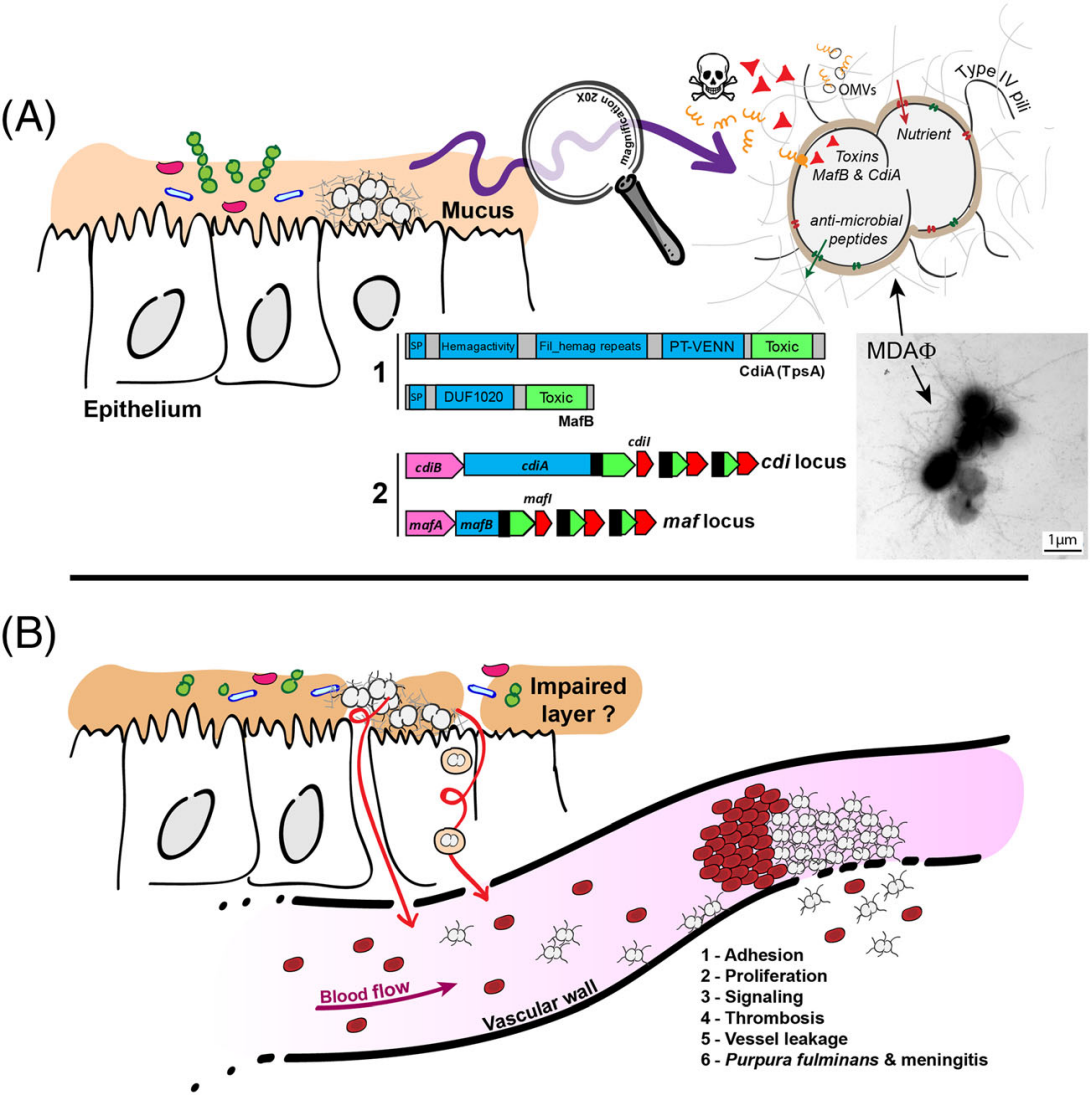
*Neisseria meningitidis*, a commensal and pathogenic bacterium. A. *N*. *meningitidis* grow in the mucus in nasopharynx where it encounters a poor nutritive medium and a rich microbiota. Meningococci survive by expressing capsule, LOS, the MtrCDE efflux pump, and factors that capture nutrients. *N*. *meningitidis* also express two families of polymorphic toxins: MafB and CdiA. **A1** depicts the domain organisation found in CdiA and MafB polymorphic toxins constituted by a conserved N‐terminal domain (blue boxes) apposed to a toxic domain in the variable C‐terminal region (green boxes). Many toxic activities have been reported for toxic domains (Zhang et al., 2012). SP, signal peptide; Hemag activity, hemagglutination activity domain, also called a “TPS domain” (PF05860); fil hemag repeats, filamentous hemagglutinin repeats (PF13332); PT‐VENN, pre‐toxin domain with a VENN motif; DUF1020, domain of unknown function 1020. **A2**: Simplified genomic organisations of *maf* and *cdi* loci. Full‐length toxin genes *cdiA* and *mafB* are depicted in blue with their extremity encoding the toxic activity depicted in green. Genes encoding immunity proteins are depicted in red (*cdiI* and *mafI*). Open Reading Frame (ORF) encoding alternative C‐terminal toxic domains of CdiA and MafB are depicted in green surrounded with dotted lines. *cdiB* encodes the dedicated transporter of CdiA toxin, whereas the role of *mafA* in MafB secretion is unknown. Black boxes indicate regions potentially involved in recombination. Left panel: aggregates harvested from the biomass covering a monolayer of FaDu cells infected by the meningococcal Z5463 strain (Bille et al., 2017) and labelled by the anti‐MDA polyclonal antibody coupled to 8 nm‐diameter gold particles. Representative picture of meningococcal MDAΦ phage‐dependant aggregates. Bar: 1 μm. B. For an unknown reason, meningococci cross the epithelial layer and enter the bloodstream where bacteria adhere to the vascular wall. Adhesive bacteria proliferate and induce an active signalling that leads to better adhesion and opening of the vascular barrier, vessel leakage, and massive thrombosis

Polymorphic toxins (PT) are multidomain secreted proteins primarily involved in competition between bacteria (Jamet & Nassif, 2015a; Jamet & Nassif, 2015b; Zhang, de Souza, Anantharaman, Iyer, & Aravind, 2012). Each family is defined by a conserved N‐terminal region and diverse C‐terminal toxic domains (e.g., nucleases, pore forming, or protein‐modifying activities). In PT systems, a gene encoding a protective immunity protein is always located immediately downstream of the toxin gene (Figure 1a). This immunity protein protects the toxin‐producing cell both from autointoxication and from toxin produced by other strains (Jamet & Nassif, 2015a; Jamet & Nassif, 2015b; Zhang et al., 2012). *N*. *meningitidis* possesses two distinct families of PTs encoded by *maf* and *tps* loci, which are not related to one another but share several common features of PT. The most obvious similarity lies in their typical gene organisation. Indeed, downstream of the full‐length toxin gene and its cognate immunity gene, there is a variable number of 5′ truncated toxin genes called CT(C‐terminal)‐cassettes, also associated with their specific immunity gene (Arenas, Schipper, van Ulsen, van der Ende, & Tommassen, 2013; Jamet et al., 2015). These alternative toxic domains could potentially allow antigenic variation by recombination with the full‐length toxin gene (i.e., *mafB* or *tpsA*; Arenas et al., 2013; Arenas et al., 2015; Jamet et al., 2015). Hence, CT‐cassettes constitute a potential reservoir of toxic domains. Strikingly, the number of CT‐cassettes and the nature of the toxic activities they encode are highly variable from one strain to another. Because CT‐cassettes are always found associated with their cognate specific immunity genes, it suggests that their repertoire of toxin‐immunity modules could determine the ability of strains to compete or cooperate with each other (Figure 1a).

The *tps* loci encode two‐partner secretion systems (TPS). An outer membrane transporter generically named TpsB allows the translocation of a very large filamentous protein (>2,000 amino acid residues) generically named TpsA (Hodak et al., 2006; ur Rahman, Arenas, Ozturk, Dekker, & van Ulsen, 2014). TpsA proteins are well‐known to carry adhesive properties as described for the filamentous hemagglutinin adhesin from *Bordetella pertussis* (Hodak et al., 2006). Several roles have been attributed to meningococcal TpsA proteins including adhesion to epithelial cells (Schmitt et al., 2007) and promotion of biofilm formation (Neil & Apicella, 2009). A TpsA protein carrying a toxic domain at its C‐terminus is usually renamed CdiA toxins (Aoki et al., 2005). CdiA toxins have been initially described in *Escherichia coli*, where they have been shown to mediate a contact‐dependent inhibition of the growth of neighbouring bacteria ((Contact‐dependent inhibitor (CDI); Aoki et al., 2005). A role in interbacterial competition has been experimentally confirmed for a *tps* locus in the meningococcal strain B16B6 belonging to the hypervirulent cc11 clone (Arenas et al., 2013). This suggests that other meningococcal TpsA proteins harbouring various CT toxic domains could constitute functional CDI systems able to mediate growth inhibition of bacteria that lack protective immunity protein.

The MafB family is restricted to the *Neisseria* genus, in contrast with CdiA and most other PT families that are found in several genera (Zhang et al., 2012). Strikingly, *maf* genes represent 2% of the genome of pathogenic *Neisseria* and are likely to play important roles for pathogenesis of this genus (Jamet et al., 2015). Maf proteins are encoded by genes belonging to the multiple adhesin family (*maf*). MafA is a putative adhesin because it has been shown to interact with a specific glycolipid found on mammalian cells (Paruchuri, Seifert, Ajioka, Karlsson, & So, 1990). There are three *maf* genomic islands with conserved chromosomic location in meningococcal genomes that harboured *mafB* and *mafI* genes (Jamet et al., 2015). Anne Jamet et al. recently demonstrated that *mafB* genes encode secreted polymorphic toxins specifically neutralised by immunity proteins encoded by cognate *mafI* genes (Jamet et al., 2015). Of note, apart from the toxic domain that can be the same in a CdiA or a MafB toxin, MafB proteins do not show any similarity with TpsA proteins. Nor there is any similarity between MafA and CdiB proteins. While MafB proteins are secreted by meningococcus, their mode of secretion remains unknown, and there is no clue of a role of MafA in MafB secretion. The presence of MafA adhesin and MafB toxins in outer‐membrane vesicles (OMVs; Zielke, Wierzbicki, Weber, Gafken, & Sikora, 2014) suggests that OMVs could be a vehicle for delivery of MafB toxins to neighbouring bacteria or even to eukaryotic cells because MafA is able to bind cellular glycolipids (Paruchuri et al., 1990). Hence, MafB toxins could have multiple roles in vivo during pathogenesis or commensalism, which remain to be deciphered.

### 2.2 The MDA filamentous phage

Filamentous bacteriophages are part of the horizontally mobile elements (Mai‐Prochnow et al., 2015). As example, CTXΦ of *Vibrio cholerae*, which encodes the cholera toxin, can transduce nontoxigenic strains into toxigenic strains. The Pf bacteriophages of *Pseudomonas aeruginosa* are involved in the formation of biofilm by inducing cell death and the subsequent release of bacterial DNA (Rice et al., 2009). Filamentous bacteriophages are also involved in horizontal gene transfer (VPIΦ of *V*. *cholerae*), increase of motility (RSS1Φ of *Ralstonia solanacearum*, SW1Φ of *Shewanella piezotolerans*) and formation of host morphotypic variants (Cf1tΦ of *Xanthomonas campestris*, Pf4Φ, and Pf6Φ of *P*. *aeruginosa*; Mai‐Prochnow et al., 2015).

Whole genomes comparison using a collection of meningococci of defined pathogenic potential allowed the identification of an 8‐kb island that was associated with invasive infections. This island, designated MDA for Meningococcal Disease Associated island, encodes a functional filamentous prophage able to produce infectious filamentous phage particles (MDAΦ; Bille et al., 2005; Bille et al., 2008). The MDAΦ particles, each about 1,200 nm long, are secreted through the type IV pilus secretin PilQ and form a mesh of long bundles of filaments anchored to meningococci (Meyer et al., 2016). To infect naive recipient strains, MDAΦ particles use type IV pili as receptor and benefit from pilus retraction to access the cytoplasm of new hosts. Interestingly, production of MDAΦ particles and type IV pili seems to be mutually exclusive. Bille et al. demonstrated that MDAΦ particles form large bundles surrounding and connecting bacteria. These bacteria‐bacteria interactions increase the biomass of encapsulated meningococci interacting with monolayer of epithelial cells (Bille et al., 2017). Altogether, these data suggest that MDAΦ increases the bacterial load at the site of entry that in turn enhances the probability of bacterial translocation into the bloodstream and/or the dissemination of the bacteria in the general population.

## 3 INVASION OF THE BLOODSTREAM FROM THE PORT OF ENTRY

The mechanisms by which meningococci leave the nasopharynx and invade the bloodstream remain unknown. Active translocation of *N*. *meningitidis* following bacterial internalisation and trafficking within intracellular vacuoles is one of the major hypothesis, especially considering that the outer membrane proteins Opa and Opc were involved in an active process of internalisation that could be followed by translocation of bacteria through cellular monolayers (Billker, Popp, Gray‐Owen, & Meyer, 2000; de Vries, van Der Ende, van Putten, & Dankert, 1996; Schmitter et al., 2007; Virji, Makepeace, Ferguson, Achtman, & Moxon, 1993). Internalisation of meningococci may also be enhanced by other bacterial factors such as NadA (Bozza et al., 2014; Montanari et al., 2012), GltT‐GltM (Takahashi, Kim, & Watanabe, 2011), AutB (Arenas et al., 2016), or interaction of porin with TLR2 host receptor (Toussi, Wetzler, Liu, & Massari, 2016). Interestingly, two studies observed that infection of fully differentiated epithelial cells (Calu‐3 cell line) restrained meningococci at the apical domain (Barrile et al., 2015; Sutherland, Quattroni, Exley, & Tang, 2010), suggesting that differentiated epithelial cells prevent the dissemination of meningococci. Further experiments using Calu‐3 cells in air‐liquid interface culture, a model in which cells are grown with the apical domain facing the air, which generates a model more morphologically representative of the airway epithelium with a more rugged apical topography and greater glycoprotein secretion (Grainger, Greenwell, Lockley, Martin, & Forbes, 2006), will be needed to address the question of physiologically relevant colonisation of epithelial cells. It should be pointed out that circumstances, such as viral infections (Hubert, Watier, Garnerin, & Richardson, 1992) or climatic conditions (Sultan, Labadi, Guegan, & Janicot, 2005) possibly responsible for damaging the airway epithelium can favour meningococcal infections.

## 4 BLOOD‐BORNE COLONISATION OF THE VASCULAR COMPARTMENT

Once in the blood, the meningococcus benefits from the same virulence factors that ensure its survival in mucus to survive and proliferate in the blood: the polysaccharidic capsule and the LOS, the factor H binding protein (fHBP), the MtrCDE efflux pump, up to four different transporters to capture iron including transferrin binding proteins, lactoferrin binding proteins, and two independent heme transport systems that recognise haemoglobin and haptoglobin‐haemoglobin (HmbR and HpuAB, respectively; Perkins‐Balding, Ratliff‐Griffin, & Stojiljkovic, 2004). The virulence factor appearing to be specific to blood infection is the type IV pilus, which is the only mean by which capsulated meningococci adhere to endothelial cells in vivo (Join‐Lambert et al., 2013)

### 4.1 The importance of endothelial cell colonisation, the Achilles' heel of meningococci

Examination of post‐mortem samples of meningococcemia has shown that *N*. *meningitidis* forms large colonies at the apical surface of capillary endothelial cells throughout the body including spleen, skin, liver, kidney, heart, and brain. Retraction of endothelial cells, capillary disruption, haemorrhages, and luminal thrombi is observed. These observations are uncommon compared with most invasive bacterial pathogens. Thus, the specific ability of *N*. *meningitidis* to colonise and specifically interact with peripheral and brain microvessels is likely responsible for its capacity to both cross the blood–brain barrier and induce a thrombotic/leakage syndrome that in severe forms lead to *purpura fulminans*. Meningococcal interaction with endothelial cells is specific of human cells. Meningococci are unable to interact with any non‐human cells, thus hampering the ability to study the mechanisms and the consequences of this interaction in vivo. Recently, a humanised model using severe combined immunodeficiency mice grafted with human skin was developed (Join‐Lambert et al., 2013). In this model, where vessels of human origin from the graft anastomose to the mice vessels, blood‐borne *N*. *meningitidis* readily adhere to human endothelial cells. Bacterial‐induced vascular damages are very similar to those described in patients, including perivascular infiltrates, thrombosis, and vascular leakage associated with massively infected vessels (Harrison et al., 2002) that ultimately lead to the death of infected grafted animals (Capel et al., 2017). In addition, vascular damages are localised only in vessels colonised by meningococci. Capel et al. showed that adhesion to human skin vessels is a prerequisite for virulence of *N*. *meningitidis* and the maintenance of a sustained bacteremia because bacteria unable to adhere to microvessels are rapidly cleared from the bloodstream (Capel et al., 2017). Altogether, this points out the essential role of microvascular colonisation in meningococcal pathogenesis (Figure 1b).

Due to the predominant role of meningococcal adhesion in pathogenesis, targeting bacterial colonisation to microvessels represents a particularly promising strategy in the treatment of invasive meningococcal infections. Recently, Denis et al. have identified a family of compounds, which promotes within minutes the loss of type IV pili (see next paragraph) and, subsequently, alter all the functions carried by these structures, including twitching motility and adherence to human endothelial cells (Denis et al., 2019). Using the humanised mouse model of skin infection, they showed that these compounds exert a strong protective effect against the pathophysiological events occurring during meningococcemia. They reduce colonisation of the human vessels by circulating meningococci and prevent subsequent vascular dysfunctions, intravascular coagulation, and overwhelming inflammation, the hallmarks of invasive meningococcal infections. Finally, in consistence with the role of the vascular niche in promoting sustained bacteraemia leading to mice lethality (Capel et al., 2017), these compounds reduce bacteraemia and increase mice survival. In association with antibiotics, they reduce vascular inflammatory and thrombotic responses that were shown to be highly detrimental and correlated with the severity of the disease in patients (Girardin, Grau, Dayer, Roux‐Lombard, & Lambert, 1988; van Deuren et al., 1995; Waage, Brandtzaeg, Halstensen, Kierulf, & Espevik, 1989).

The identified molecules (trifluoperazine and thioridazine) belong to phenothiazines, a family of compounds previously used in human medicine to treat psychotic disorders. Due to the well‐conserved set of proteins involved in type IV pilus biosynthesis in Gram‐negative bacteria, interfering with Tfp‐mediated host cell interaction by these phenothiazines represents an attractive strategy to modify the course of diseases induced by piliated bacterial pathogens. Data strongly suggest that these phenothiazines target the sodium pumping NADH:ubiquinone oxidoreductase complex (Na^+^‐NQR). This protein complex, highly conserved among numerous nonpathogenic and pathogenic bacteria, creates a sodium motive force through the translocation of Na^+^ across the inner cell membrane. In many pathogenic bacteria, Na^+^ gradient is an entry site for electrons into the respiratory chain toward ATP synthesis or to sustain ionic homeostasis, nutrient transports, flagellum rotation, and other essential processes (Juarez & Barquera, 2012; Reyes‐Prieto, Barquera, & Juarez, 2014). Interestingly, meningococcal isogenic mutants, devoid of individual *nqr* genes were poorly piliated and aggregative, thus linking piliation and NQR‐mediated Na^+^ pumping activity. How this sodium gradient affects type IV pilus dynamics remains to be explored.

### 4.2 Type IV pili and their interaction with human cell receptors

Tfp are long and dynamic polymeric fibres made of pilin monomers (Egelman, 2017; Kolappan et al., 2016). This fibre is assembled from a platform in the inner membrane (PilM,N,O,P,G). Two ATPases permit the elongation (PilF) and the retraction (PilT) of the fibre that protrudes through the secretin PilQ (Hospenthal, Costa, & Waksman, 2017). The fibre is composed of the monomeric core pilin PilE and of three minor pilins that are responsible for Tfp‐associated phenotypes. ComP is responsible for natural competence for DNA transformation (Brown, Helaine, Carbonnelle, & Pelicic, 2010; Cehovin et al., 2013); PilV is involved in adhesion and signalling to human cells (Bernard et al., 2014; Mikaty et al., 2009), and the minor pilin PilX is essential to promote interbacterial interactions, a process named aggregation (Helaine et al., 2005; Helaine, Dyer, Nassif, Pelicic, & Forest, 2007). Aggregation of bacteria is allowed by the interaction between antiparallel pili and is a key aspect of colonisation. Indeed, a PilX‐defective mutant is piliated and unable to form aggregates or colonies on the top of endothelial cells. This interaction between antiparallel pili also exerts forces on both fibres that are responsible for a transition between two conformations: a packed and an elongated conformation. This transition reveals new epitopes, previously buried into the fibre and involved in the interaction with cellular receptors (see below; Biais, Higashi, Brujic, So, & Sheetz, 2010; Brissac, Mikaty, Dumenil, Coureuil, & Nassif, 2012; Kolappan et al., 2016). Furthermore, the dynamic of bacterial aggregates is key during colonisation of the blood capillary network. Bonazzi et al. recently revealed that the ATPase PilT drives adaptation of aggregates to tubular geometry found in capillaries, a property necessary for colonisation of blood vessels (Bonazzi et al., 2018).

Initial attachment of *N*. *meningitidis* to endothelial cells requires the interaction of tfp with human cell receptors. Several receptors were proposed as being able to interact with Tfp: the complement regulatory protein CD46, the Laminin receptor, or the platelet activating factor receptor (Jen et al., 2013; Kallstrom, Liszewski, Atkinson, & Jonsson, 1997; Kirchner, Heuer, & Meyer, 2005; Orihuela et al., 2009). During the last decade, CD147 (also known as Basigin/Emmprin) has been identified as an important adhesion receptor on both brain and peripheral endothelial cells. CD147 is a member of the immunoglobulin (Ig) superfamily comprising two Ig‐like domains (Iacono, Brown, Greene, & Saouaf, 2007). CD147 is massively recruited at sites of *N*. *meningitidis* adhesion. This recruitment precedes cytoskeletal rearrangement specific of meningococcal signalling onto endothelial cells (Carbonnelle et al., 2009). Bernard et al. showed that CD147 directly interacts with recombinant PilE and PilV, in contrast to other minor pilins: PilX and ComP (Bernard et al., 2014). Interestingly, CD147 is associated in a preformed complex with the α‐actinin‐4 scaffolding protein and the β2‐adrenergic receptor (β2AR; family of G protein coupled receptor [GPCR]). This pre‐existing association facilitates the activation of the β2AR by the two pilins PilE and PilV that activate and amplify a βarrestin‐dependent signalling pathway leading to the enrichment of ERM proteins. These proteins anchor the actin network at the site of bacterial adhesion in a structure named cortical plaque (Coureuil et al., 2010; Maissa et al., 2017; Merz, Enns, & So, 1999; Slanina, Hebling, Hauck, & Schubert‐Unkmeir, 2012). Actin polymerisation is the consequence of the local recruitment of ErbB2, small Rho GTPases, and that of the Src tyrosine kinase activation. This cell signalling is responsible for the elongation of host‐cell membrane protrusions that stabilise bacterial colonies at the surface of blood vessels (Coureuil et al., 2010; Lambotin et al., 2005; Mikaty et al., 2009). Besides, it has been recently proposed that the interaction of Tfp with host‐cell plasma membrane triggers its remodelling as discrete and dynamic protrusions in a process reminiscent of membrane wetting (referred to as 1D‐wetting; Charles‐Orszag et al., 2018). The authors suggest that membrane 1D‐wetting drives subsequent actin polymerisation and recruitment of cortical plaque associated proteins. In this context, the exact role of 1D‐wetting in β2AR‐induced remodelling of the apical surface is not clear; all the more since the involvement of this former phenomenon in meningococcal‐induced signalling has not been formally proven.

The nature of the epitope targeted by meningococcal Tfp on their cellular receptors is still unknown. To date, studies aiming at demonstrating receptor‐pilin interaction never revealed how both partners interact with each other. Some evidences suggested that Tfp from other pathogens interact with host cell carbohydrates. For instance, Tfp of *Pseudomonas aeruginosa* bind complex N‐glycans (Bucior, Pielage, & Engel, 2012) and that of *Vibrio parahaemolyticus* interact with chitin, a long‐chain polymer of N‐acetylglucosamine (Frischkorn, Stojanovski, & Paranjpye, 2013). A recent study by Mubaiwai et al. revealed that Tfp of the meningococcal strain MC58 can interact with complex N‐glycan such as GD2 ganglioside, a glycan composed of a core GalNAcβ1‐4Galβ1‐4Glc, and a terminal sialic acid (Neu5Ac; Mubaiwa et al., 2017). However, this glycan is only expressed in the cerebellum and peripheral nerves in humans and cannot account for the ability of meningococci to colonise human vessels (Ahmed & Cheung, 2014). Another open question is the role of the mechanical forces applied by Tfp. Indeed, using optical tweezers, Biais et al. showed that retraction of a single pilus generates forces up to 110 pN. Bundles of Tfp, which result from the association of eight to 10 pili, act as coordinated retractable units. Thus, bundles can generate retraction forces in the nanonewton range (Biais, Ladoux, Higashi, So, & Sheetz, 2008). Interestingly, some GPCRs were described as mechanosensors and, as such, may sense membrane tension. For instance, angiotensin II type 1 receptor is capable of sensing membrane stretching (Wang, Hanada, Gareri, & Rockman, 2018) and parathyroid hormone type 1 receptor senses mechanochemical signals in preosteoblatic cells (Zhang, Frangos, & Chachisvilis, 2009). The possibility that CD147 and/or the β2AR can be involved in mechanosensing of Tfp pulling forces needs to be further studied.

### 4.3 Consequences of blood vessels colonisation

A major consequence of bacterial adhesion to endothelial cells is the loss of vessels integrity. *N*. *meningitidis* causes vascular leakage through several parallel pathways that ultimately lead to the disassembly of both adherens and tight junctions, which is likely to be responsible in vivo for the peripheral leakage syndrome and in the brain for the crossing of the blood–brain barrier. Following bacterial adhesion onto endothelial cells, the adherens‐junction protein VE‐cadherin is relocated at sites of bacterial adhesion via the mislocalization of the Par3/6 polarity complex (Coureuil et al., 2009). Besides, *N*. *meningitidis* also promotes the cleavage of occludin (a component of the tight junctions) by the metalloproteinase MMP‐8, further altering the sealing of intercellular junctions (Schubert‐Unkmeir et al., 2010). In addition, *N*. *meningitidis* activates other signalling events in endothelial cells, but their exact consequences on endothelium integrity have not been assessed yet. Meningococci induce host‐cell calcium release from intracellular stores (Asmat, Tenenbaum, Jonsson, Schwerk, & Schroten, 2014); p21 and cyclin G2‐dependent cell cycle arrest at S phase (Oosthuysen, Mueller, Dittrich, & Schubert‐Unkmeir, 2016); Acid sphingomyelinase (ASM) and ceramide exposure in ErbB2 containing domain at plasma membrane (Simonis, Hebling, Gulbins, Schneider‐Schaulies, & Schubert‐Unkmeir, 2014).

Another major consequence of meningococcal interaction with endothelial cells is an increase of the procoagulant activity of the endothelium. Indeed, meningococcemia is very often complicated by thrombosis events. Up to 70% of patients develop a cutaneous purpura that reflects skin microvascular thrombosis and red blood cell extravasation (Thompson et al., 2006). Moreover, about 25% of the patients develop a *purpura fulminans* syndrome that associates extensive cutaneous thrombosis, ischemic necrosis of skin and organs (such as the adrenal glands), and a severe septic shock (Powars et al., 1993). All these thrombotic events are far more common in meningococcemia than in any other bacterial infections, suggesting that meningococci induce a specific dysregulation of coagulation. The humanised mouse model of meningococcal vascular colonisation described above has unveiled an important step in thrombosis development during meningococcemia: In this model, thrombosis was specifically associated with meningococcal adhesion to human endothelial cells. These results suggest that meningococcal interaction with endothelial cells is a key contributor to thrombosis. While meningococci express common prothrombotic factors (such as the LOS that mediates tissue factor expression [Mirlashari, Hoiby, Holst, & Lyberg, 2001; Ovstebo et al., 2012; Schlichting, Lyberg, Solberg, & Andersen, 1993]), a recent work has demonstrated that meningococcal Tfp‐mediated adhesion on endothelial cells specifically induces the activation of a membranous protease, a disintegrin metalloprotease 10, which subsequently cleaves the endothelial protein C receptor (EPCR) in a process known as shedding. This shedding is detrimental in the context of meningococcemia. Indeed, EPCR normally binds Protein C (PC), and this binding accelerates the rate of PC activation. Activated Protein C (aPC) is a potent anticoagulant that cleaves several coagulation factors and by such it prevents any overwhelming coagulation activation. An acquired or congenital severe deficit in PC is per se a cause of non‐infectious *purpura fulminans*, demonstrating the key role of this protein in preventing this syndrome. Hence, meningococcal Tfp‐mediated adhesion on endothelial cells, by inducing EPCR shedding, impairs the aPC negative feedback on coagulation (Lecuyer et al., 2018). So far, such dysregulation of coagulation system seems to be specific to the meningococcus. Besides, fibrinolysis that ends with fibrin clot destruction is also impaired during meningococcal infection. Indeed, meningococcal LOS induces the release of the fibrinolysis inhibitor plasminogen activator inhibitor by monocytes, and this certainly contributes to the extensive microvascular thrombosis (Mirlashari et al., 2001; Schlichting et al., 1993).

## 5 CONCLUSION

Many studies aiming at understanding how *N*. *meningitidis* colonise the pharyngeal niche, disseminate, and colonise blood vessels have been carried out and gave a clear overview of *N*. *meningitidis* interaction with epithelial and endothelial cells, providing a promising route to the treatment of invasive meningococcal diseases. However, so far, the lack of suitable models has limited our understanding of meningococcal colonisation of the upper airway or that of the opening of the blood–brain barrier. The understanding of the “colonisation phase” of *N*. *meningitidis* is a prerequisite to addressing the dissemination route responsible for bloodstream dissemination.
